# Trajectories of Cardiac Function Following Treatment With an Impella Device in Patients With Acute Anterior ST-Elevation Myocardial Infarction

**DOI:** 10.1016/j.cjco.2022.11.002

**Published:** 2022-11-05

**Authors:** Gregorio Tersalvi, Adrian Attinger-Toller, Dhanya Kalathil, Dario Winterton, Giacomo Maria Cioffi, Mehdi Madanchi, Thomas Seiler, Marc Stadelmann, Francesca Goffredo, Patrick Fankhauser, Federico Moccetti, Mathias Wolfrum, Stefan Toggweiler, Andreas Bloch, Richard Kobza, Florim Cuculi, Matthias Bossard

**Affiliations:** a Cardiology Division, Heart Centre, Luzerner Kantonsspital, Lucerne, Switzerland; b Department of Health Sciences and Medicine, University of Lucerne, Lucerne, Switzerland; c Department of Anesthesia, Critical Care, and Pain Medicine, Massachusetts General Hospital and Harvard Medical School, Boston, Massachusetts, USA; d Department of Intensive Care Medicine, Luzerner Kantonsspital, Lucerne, Switzerland

## Abstract

**Background:**

Left ventricular (LV) unloading via the percutaneous micro-axial Impella pump is increasingly used in patients with anterior ST-segment elevation myocardial infarction (STEMI) and overt cardiogenic shock. In this context, the evolution of cardiac function and dimensions beyond hospital discharge remains uncertain. We aimed to characterize echocardiographic changes over time in patients with acute anterior STEMI treated with an Impella device.

**Methods:**

From an ongoing prospective registry, consecutive patients with acute anterior STEMI managed with an Impella device were extracted. Transthoracic echocardiography was performed at index hospitalization and at first outpatient follow-up. Predictors of response, defined as a ≥ 10% absolute increase in left ventricular ejection fraction (LVEF) at follow-up, were sought.

**Results:**

A total of 66 patients (89.4% male, aged 64.3 ± 11.6 years) with anterior STEMI were treated with an Impella device in the first 24 hours of hospitalization, from 2014 to 2022. In-hospital mortality was 24%. Major bleeding and vascular complications requiring surgery occurred in 24% and 11% of patients, respectively. At baseline, mean LVEF was 34% ±12%. At follow-up, survivors showed a significant increase in LVEF (*P* < 0.0001), whereas LV dimensions, diastolic parameters, and measures of right ventricular dimension and function remained stable. Overall, 28 patients had a ≥ 10% absolute increase in LVEF at follow-up. Baseline creatinine was the only significant predictor of response at univariate analysis.

**Conclusions:**

Among patients with anterior STEMI requiring mechanical hemodynamic support with an Impella device, the majority of survivors showed a sustained increase in LV function, without evidence of adverse remodelling. This signal warrants further investigation in dedicated trials.

## Graphical abstract

**Figure:**
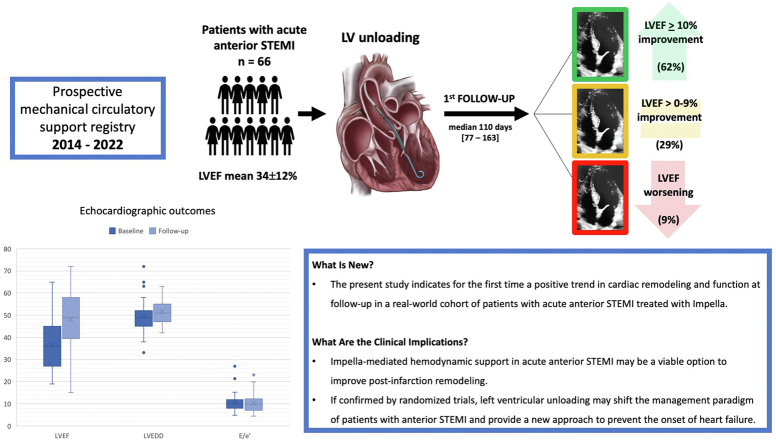

## Résumé

**Contexte:**

La décharge du ventricule gauche (VG) à l’aide de la pompe microaxiale percutanée Impella est de plus en plus employée chez les patients qui présentent un infarctus du myocarde avec élévation du segment ST (STEMI) et un choc cardiogénique manifeste. Dans ce contexte, l’évolution de la fonction et des dimensions cardiaques après le congé de l’hôpital demeure incertaine. Nous avons cherché à caractériser les variations des paramètres échocardiographiques au fil du temps chez les patients ayant subi un STEMI antérieur aigu traités au moyen d’un dispositif Impella.

**Méthodologie:**

À partir d’un registre prospectif actif, nous avons extrait les dossiers de patients consécutifs ayant subi un STEMI antérieur aigu et pris en charge au moyen d’un dispositif Impella. Une échocardiographie transthoracique a été effectuée durant l’hospitalisation de référence puis à la première visite de suivi ambulatoire. Les facteurs prédictifs de la réponse, définis comme une augmentation absolue ≥ 10 % de la fraction d’éjection ventriculaire gauche (FEVG) au moment du suivi, ont été recherchés.

**Résultats:**

Au total, 66 patients (89,4 % d’hommes, âgés de 64,3 ± 11,6 ans) ayant subi un STEMI antérieur ont été traités au moyen d’un dispositif Impella pendant les 24 premières heures de l’hospitalisation, entre 2014 et 2022. Le taux de mortalité hospitalière était de 24 %. Des hémorragies majeures et des complications vasculaires ayant nécessité une intervention chirurgicale sont survenues chez 24 % et 11 % des patients, respectivement. Au début de l’étude, la FEVG moyenne était de 34 % ± 12 %. Lors du suivi, les survivants affichaient une augmentation significative de la FEVG (*p* < 0,0001), alors que les dimensions du VG, les paramètres diastoliques et les mesures de la taille et de la fonction du ventricule droit étaient demeurés stables. Globalement, 28 patients affichaient une augmentation absolue de la FEVG ≥ 10 % lors du suivi. D’après l’analyse univariée, la créatininémie initiale était le seul facteur prédictif important de la réponse.

**Conclusions:**

Parmi les patients ayant subi un STEMI antérieur nécessitant un support mécanique hémodynamique au moyen d’un dispositif Impella, une augmentation durable de la fonction ventriculaire gauche, sans signe de remodelage indésirable, a été observée chez la majorité des survivants. Ce signal justifie une évaluation plus approfondie dans le cadre d’essais cliniques conçus à cet effet.

Patients presenting with acute anterior ST-segment myocardial infarction (STEMI) and beginning or overt cardiogenic shock still show a very high morbidity and mortality rate.^1^^,^^2^ Considering the rapid and potentially lethal downward spiral of myocardial ischemia and related systemic organ dysfunction, early identification and treatment are crucial to increase chances of survival.

The contemporary management of STEMI-associated cardiogenic shock focuses on treating the underlying cause by prompt revascularization of the culprit vessel,^3^ as well as hemodynamic stabilization by means of pharmacologic therapy, mechanical ventilation, and mechanical circulatory support (MCS) in patients who are not responding to standard treatment.^2^

The Impella device (Abiomed, Danvers, MA) provides percutaneously delivered MCS by unloading the left ventricle (LV) by pumping blood from the LV to the aorta.^4^^,^^5^ As a result, the Impella device reduces LV end-diastolic volume and pressure, leading to a reduction of myocardial wall tension and workload, both of which diminish myocardial oxygen demand.^5^^,^^6^

Experimental studies suggest that early LV unloading in the setting of an acute myocardial infarction (MI) reduces infarct size.^7^, ^8^, ^9^ However, the impact of ventricular unloading with the Impella device on cardiac remodelling in patients with acute anterior STEMI complicated by cardiogenic shock is still largely unknown.

The purpose of the present study is to describe the time course of cardiac function and remodelling after acute anterior STEMI treated with the Impella device.

## Methods

### Study population and data collection

From an ongoing MCS registry (ClinicalTrials.gov identifier: NCT04117230), we analyzed consecutive patients older than 18 years of age at the Heart Centre of the Luzerner Kantonsspital, Lucerne, Switzerland, which represents the tertiary cardiology facility of Central Switzerland, between December 2014 and February 2022.

The inclusion criteria were as follows: (i) acute (defined as onset of symptoms within 24 hours) anterior STEMI; (ii) implantation of a left-heart Impella device within 24 hours from STEMI diagnosis. Patients presenting with non-anterior STEMI localization, as well as subacute STEMI (ie, time from pain onset to diagnosis > 24 hours), and those receiving another type of MCS, were excluded. All patients were treated according to current STEMI guidelines—thus, they received a coronary angiography and a primary percutaneous coronary intervention (PCI) to the culprit vessel.^1^^,^^10^ All baseline coronary angiograms were independently analyzed by 2 senior invasive cardiologists (A.A.-T. and M.B.). Data on demographics, previous history, cardiovascular risk factors, angiography, laboratory tests, and discharge medications were collected by study personnel and entered in a dedicated database on REDCap (for research electronic data capture, version 11.1.27).^11^ Prospective data acquisition after enrollment was approved by the local and national ethics committee (EKNZ/ Swissethics, BASEC-ID 2019-00274) and conducted according to the principles of the Declaration of Helsinki.

### Impella device implantation

The catheter-based, Impella 2.5 and CP continuous microaxial flow pumps can be implanted fully percutaneously, generally through the femoral artery.^12^ The indication for Impella device implantation was determined on a case-by-case basis by the treating invasive cardiologist performing the coronary angiography.

As part of our clinical routine, we obtained a contrast angiogram in an ipsilateral projection to assess puncture height and anatomic suitability of the iliac and femoral arteries prior to Impella device insertion. Ultrasound guidance was used, whenever possible. All patients were anticoagulated with unfractionated heparin to achieve an activated clotting time > 250 seconds during PCI. Finally, the Impella devices were inserted over a stiff 0.018-inch guide wire, advanced under fluoroscopy, and positioned in a retrograde fashion across the aortic valve.

The timing of Impella device insertion generally followed 2 principles, as follows: (i) implanting the device and establishing LV unloading prior to PCI, and ensuring continuous support during and after PCI. This approach is preferred for patients presenting with established and profound cardiogenic shock. (ii) Implanting the device after primary PCI, a strategy generally pursued in patients showing growing inotrope-dependency, deteriorating hemodynamics, and/ or signs of multiorgan dysfunction secondary to advancing cardiogenic shock, which worsens following revascularization. In all patients, the decision for Impella device removal was made after interdisciplinary evaluation by the interventional cardiologists and critical care physicians, based on the following 3 main criteria: (i) hemodynamic support no longer needed after appropriate weaning; (ii) escalation of support (ie, extracorporeal membrane oxygenation, left ventricular assist device [LVAD], or heart transplantation); and (iii) withdrawal of therapy and/or patient death.

### Echocardiography

Transthoracic echocardiography (TTE) was performed at index hospitalization (baseline) and at outpatient follow-up. Baseline echocardiography followed the standard protocol in our centre and was performed by a board cardiologist in either the intensive care unit (Affinity or CX50, Philips Healthcare, Best, The Netherlands) or our echocardiography laboratory (EPIQ 7, Philips Healthcare or VIVID E95, GE Healthcare, Chicago, IL). If more than one TTE was performed during hospitalization, only the first one after STEMI diagnosis was taken into consideration for the analysis. Follow-up echocardiograms were performed either in our outpatient clinic or by external cardiologists. All echocardiograms were reviewed offline using a dedicated software package (Intellispace, Philips Healthcare) by an independent physician certified in TTE by the European Association of Cardiovascular Imaging, and echocardiographic measures were performed according to this institution’s guidelines.^13^

### Statistical analysis

Variables are presented as mean ± standard deviation or median (1st-3rd quartile), depending on their distribution. Normality of distribution was assessed using the Shapiro-Wilk test and a Q-Q plot. Categorical variables are presented as number (percentage). Continuous variables were compared using the Student *t* test or the Wilcoxon signed-rank test, depending on distribution. Categorical variables were compared using Fisher’s exact test. In patients who survived and had echocardiographic follow-up within the first months after discharge, we compared baseline and first follow-up echocardiographic data. According to previous literature, which showed a left ventricular ejection fraction (LVEF) absolute increase ≥ 10% after MI being prognostically significant,^14^ we stratified patients based on LVEF improvement at follow-up. After dividing patients into groups of “responders” (difference in LVEF between first follow-up and baseline ≥ 10%) and “nonresponders,” we compared the 2 groups for variables known to be associated with prognosis. We performed no imputation for missing data. Variables were compared when < 20% of data were missing. Two-sided *P* values l< 0.05 were considered statistically significant. JMP Pro (Version 16, SAS Institute, Cary, NC) was used for data quality assessment, statistical analysis, and graphical representation.

## Results

**Figure 1:**
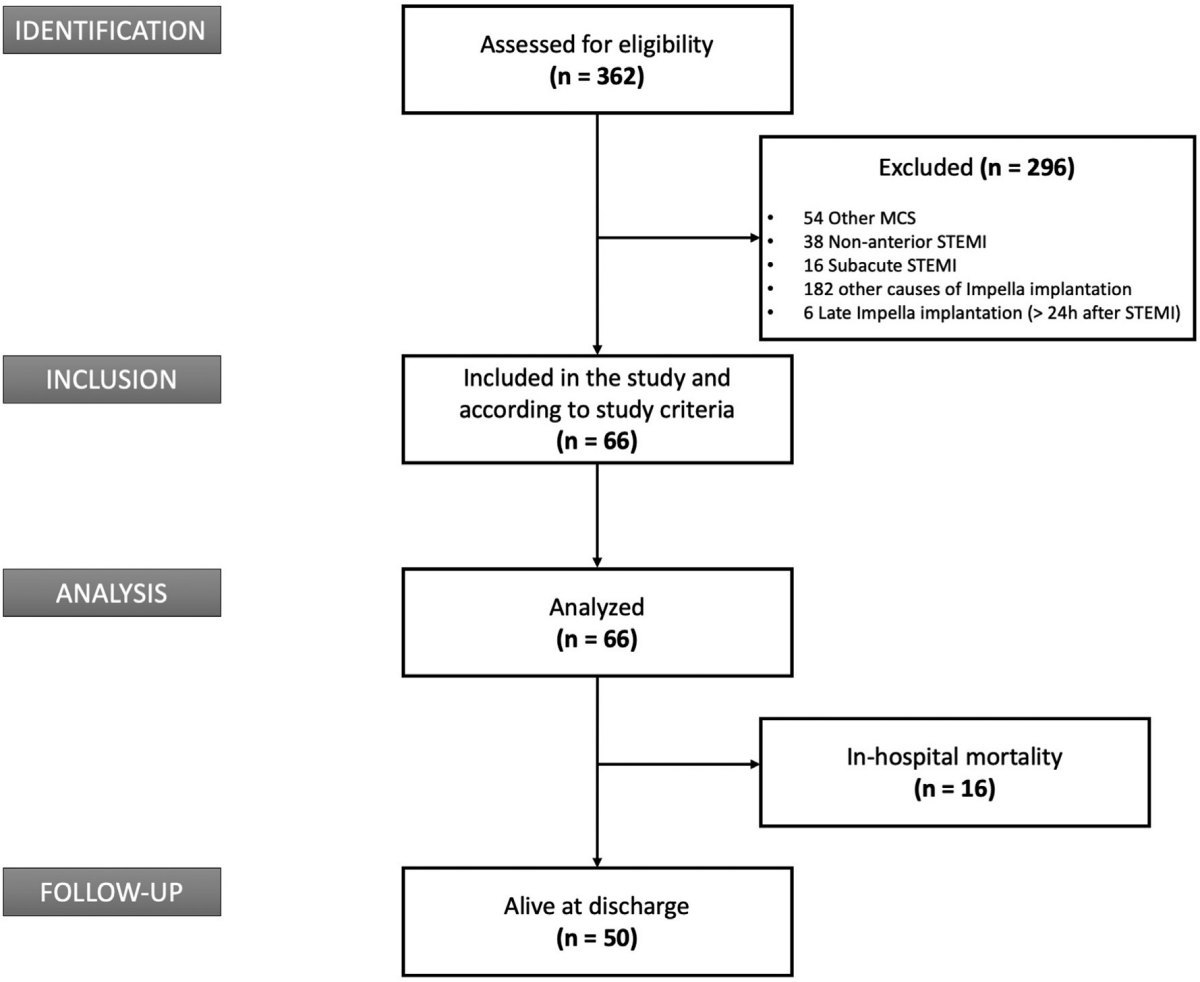
**St**rengthening the **R**eporting of **Ob**servational Studies in **E**pidemiology (STROBE) study flowchart. MCS, mechanical circulatory support; STEMI, ST-elevation myocardial infarction.

Overall, 66 consecutive patients with the prespecified inclusion criteria were included. Those patients had been treated between December 2014 and February 2022. The patient inclusion flowchart is shown in Figure 1.

### Baseline characteristics

The baseline characteristics are displayed in Table 1 and Supplemental Table S1. Our cohort’s mean age was 64 ± 12 years; 89% were male. Known coronary artery disease was present in 18% of patients. Among cardiovascular risk factors, smoking (47%) and arterial hypertension (52%) were common. Approximately one-third of all patients presented with out-of-hospital cardiac arrest, and 79% were in profound cardiogenic shock (Society for Cardiovascular Angiography and Intervention [SCAI] class C, D, or E).

**Table 1:** Baseline characteristics

Characteristic	All patients (n = 66)
Age, y, mean ± SD	64.3 ± 11.6
Male	59 (89.4)
BMI, kg/m^2^, median [IQR]	24.9 [24.2–28.1]
Comorbidities	
CAD	12 (18.2)
Previous MI	9 (13.6)
Previous PCI	10 (15.2)
Previous CABG	1 (1.5)
Heart failure	2 (3.0)
Atrial fibrillation	2 (3.0)
Previous stroke	3 (4.5)
PAD	7 (10.6)
CKD (eGFR < 30 ml/min per 1.73 m^2^)	3 (4.5)
Cardiovascular risk factors	
Smoking	31 (47.0)
Arterial hypertension	34 (51.5)
Dyslipidemia	25 (37.9)
Diabetes mellitus	8 (12.1)
Family history of CAD	11 (16.6)
Clinical presentation	
OHCA	25 (37.3)
Profound cardiogenic shock∗	52 (78.8)
SCAI class at admission	
A	12 (18.2)
B	2 (3.0)
C	8 (12.1)
D	19 (28.8)
E	25 (37.9)
GRACE score, points, mean ±SD	182 ± 25
CardShock score, points, median [IQR]	3 [2–4]

Values are n (%), unless otherwise indicated.

BMI, body mass index; CABG, coronary artery bypass graft surgery; CAD, coronary artery disease; CKD, chronic kidney disease; COPD, chronic obstructive pulmonary disease; eGFR, estimated glomerular filtration rate; GRACE, Global Registry of Acute Coronary Events; MI, myocardial infarction; OHCA, out-of-hospital cardiac arrest; PAD, peripheral artery disease; PCI, percutaneous coronary intervention; SCAI, Society for Cardiovascular Angiography and Intervention.

∗ Defined as SCAI class C, D, or E.

### In-hospital management and clinical outcomes

In-hospital management is summarized in Table 2 and Supplemental Table S2. Except for one patient who died before angioplasty, all patients were treated with primary PCI. No patient received thrombolysis. Most patients (96%) were implanted with an Impella CP device. The median duration of Impella device support was 33 [IQR: 23; 51] hours. Six patients received a second device, an intra-aortic balloon pump (IABP) through the contralateral femoral artery in all cases, in the first hours or days after Impella device implantation. Among these patients, 5 received concomitant use of an Impella device and an IABP, and in one patient, the Impella was explanted from a previous IABP implantation. Thirty-three patients (50%) required mechanical ventilation. The median stay in the intensive care unit was 4 (range: 2-10) days. In-hospital mortality was 24% (n = 16), mainly due to cardiac causes (ie, refractory cardiogenic shock or resuscitation). The outcomes are summarized in Table 3.

**Table 2:** In-hospital management

Variable	All patients (n = 66)
Type of support	
Impella 2.5	3 (4.5)
Impella CP	63 (95.5)
Timing of support	
Before PCI	49 (74.2)
After PCI	16 (24.2)
Indication for Impella device	
Cardiac arrest	37 (56.1)
Profound cardiogenic shock (at least SCAI class C)	63 (95.5)
Beginning cardiogenic shock (SCAI class B)	3 (4.5)
Other MCS device	
Impella device and IABP combined	5 (7.6)
IABP after Impella device explantation	1 (1.5)
LVEDP at implantation, mmHg	31.7 ± 9.5
MAP at implantation, mmHg	70 [59.0–90.0]
Ongoing resuscitation during implantation	9 (13.6)
Duration of support, h	32.7 [23–50.5]
PCI performed	65 (98.5)
Culprit vessel	
LM	13 (19.7)
LAD	53 (80.3)
ICU stay, d	4 [2–10]
Mechanical ventilation	33 (50.0)
Length of mechanical ventilation, h	90 [32.0–216.0]
Inotropes/vasoactive drugs	48 (76.2)
Duration of inotropes/vasoactives, h	57 [17.8–137.5]
New-onset AKI	12 (19.0)
Laboratory values	
Hemoglobin at admission, g/L	137 [124.5–147]
Leukocytes at admission, G/L	12.6 [10.0–16.5]
Lactate at implantation, mmol/L	2.4 [1.6–4.4]
Creatinine at implantation, μmol/L	83 [70–103]
Peak creatinine, μmol/L	107 [89.5–154.5]
Troponin T at admission, ng/L	200 [48.5–1872]
Peak troponin T, ng/L	9820 [4082–18,477.5]
Peak creatine kinase, U/L	5156 [2450.8–6853.5]
ALT, U/L	64 [30.5–158]

Values are n (%), mean ± standard deviation, or median [interquartile range].

AKI, acute kidney injury; ALT, alanine aminotransferase; IABP, intra-aortic balloon pump; ICU, intensive care unit; LAD, left anterior descending artery; LM, left main; LVEDP, left ventricular end-diastolic pressure; MAP, mean arterial pressure; MCS, mechanical circulatory support; PCI, percutaneous coronary intervention; SCAI, Society for Cardiovascular Angiography and Intervention.

**Table 3:** In-hospital outcomes

Outcome or cause	All patients (n = 66)
Outcomes	
Discharged to home	19 (28.8)
Discharged to rehabilitation	26 (39.4)
Discharged to nursing home	1 (1.5)
Transferred to other hospital	4 (6.1)
Died	16 (24.2)
Causes of in-hospital death	
Cardiac causes	9 (13.6)
Therapy withdrawal because of neurologic prognosis	3 (4.5)
Other	4 (6.1)

Values are n (%).

We observed 7 major vascular complications requiring vascular surgery, and a fairly high rate of major bleeding, most of which was access-related bleeding. Supplemental Table S3 lists all complications.

The narratives relating to patients facing in-hospital death are shown in Supplemental Table S4. Therapy at hospital discharge is displayed in Supplemental Table S5. A total of 5 patients (8%) required implantation of a cardiac defibrillator during follow-up. In all, 17 patients (26%) had died after 6 months of follow-up. One patient received a LVAD after 1 month, and one patient underwent heart transplantation 2 months after index presentation.

### Cardiac recovery by echocardiography

Echocardiography at baseline was performed in the first days after hospital admission (median 1.0 days [range: 0.4-4.5]). Overall, the LVEF was reduced (mean 34% ± 12%), whereas the left ventricular end-diastolic diameter, E/e’, and parameters of right ventricular dimension and function were within normal ranges.

The echocardiographic follow-up was performed at a median of 110 days (IQR: 77-163) after Impella device treatment. Follow-up echocardiography was unavailable in 3 patients, whereas 2 patients received a LVAD or heart transplantation. Compared to baseline, LVEF at follow-up showed a significant increase, to 48% ± 13% (*P* < 0.0001; Supplemental Fig. S1), whereas left ventricular diastolic diameter showed a statistically, but not clinically, significant increase from 49 mm (range: 45-52] to 51 mm (range: 47-55; *P* = 0.001; Supplemental Fig. S2). The other echocardiographic parameters remained unchanged (Table 4; Supplemental Fig. S3). Echocardiographic parameters of non-survivors are displayed in Supplemental Table S6.

**Table 4:** Echocardiographic parameters at baseline∗ and first follow-up†

Parameter	All patients (n = 66)	Survivors (n = 50)	First follow-up	*P* value‡
LVEF, %	34 ± 12	36 ± 11	48 ± 13	**< 0.0001**
LVEDD, mm	49 [45–52]	48 [45.0–52.0]	51 [47–55]	**0.001**
LVEDDi, mm/m^2^	25.6 ± 4.0	25.5 ± 4.1	26.5 ± 3.3	**0.001**
E/e’	9.6 [8.0–11.9]	9.6 [8.0–11.6]	9.3 [6.9–12]	0.32
LAVi, ml/m^2^	28 [23–42]	28 [23–43]	29 [21–36]	0.86
TAPSE, mm	22 [19–24]	22 [19–24]	22 [20–26]	**0.03**
RV S’, cm/s	14.6 ± 4.2	15.6 ± 4.2	13.9 ± 3.6	0.22
TR Vmax, m/s	2.8 [2.5–3.5]	2.8 [2.4–2.8]	2.4 [2.2–3.0]	0.09
RVEDAi, cm^2^/m^2^	9.4 [7.5–11.5]	9.2 [7.0–10.5]	8.5 [7.3–10.4]	0.25

Values are mean ± standard deviation or median [interquartile range]. Boldface indicates significance.

LAVi, left atrial volume index; LVEDD, left ventricular diastolic diameter; LVEDDi, left ventricular diastolic diameter index; LVEF, left ventricular ejection fraction; RVEDAi, right ventricular end-diastolic area index; RV S’, right ventricular systolic excursion velocity; TAPSE, tricuspid annular plane systolic excursion; TR Vmax, tricuspid regurgitation peak jet velocity.

∗ Median: 0.95 days [0.4 – 4.5] after admission.

† Median: 109.5 days [76.8 – 163.45] after device implantation.

‡ Survivors at baseline compared to first follow-up.

**Figure 2:**
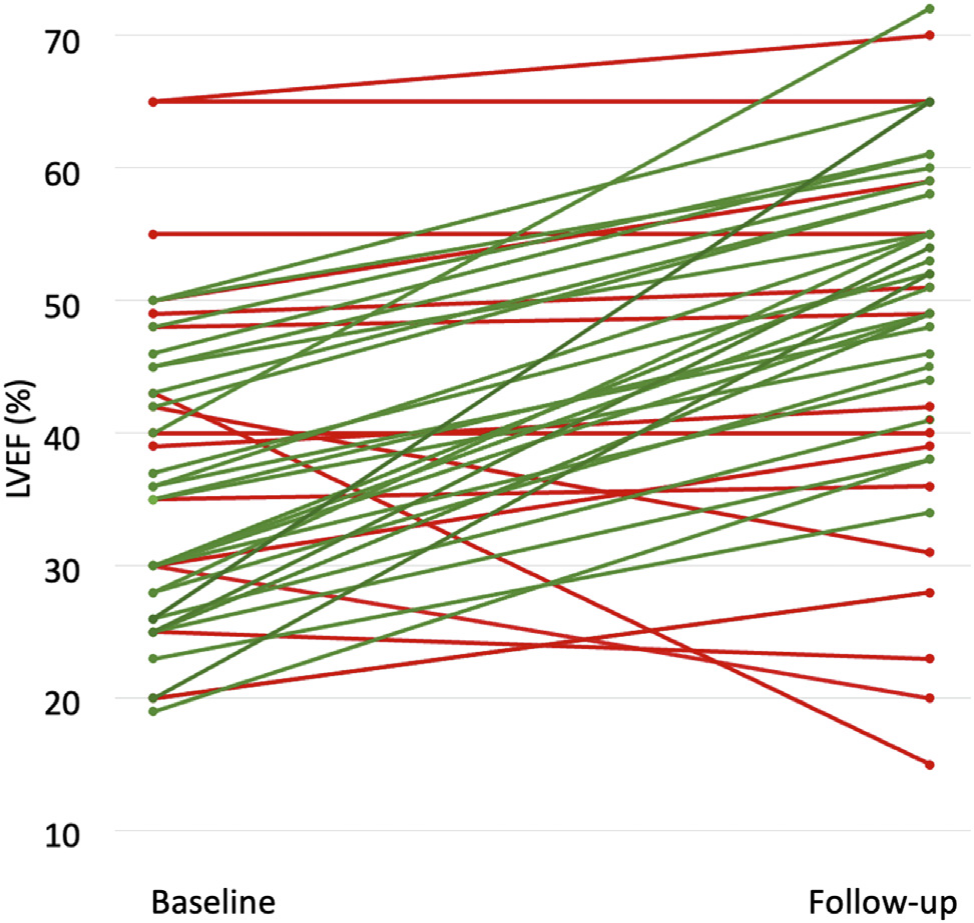
Trajectory of left ventricular ejection fraction (LVEF) from baseline (median: 0.95 days [range: 0.4-4.5] after admission) to first follow-up (median: 109.5 days [range: 76.8-163.45] after device implantation). **Green** indicates patients with a ≥ 10% absolute increase of LVEF at follow-up. **Red** indicates patients without a ≥ 10% absolute increase of LVEF at follow-up.

A total of 28 patients (62%) were identified as being responders (ie,having a ≥ 10% absolute increase of LVEF at follow-up), whereas 17 patients (38%) were identified as being nonresponders (Fig. 2). Of these, 13 (29%) showed a modest LVEF improvement (absolute increase between 0% and 9%), and 4 (9%) showed a worsening LVEF.

The performed univariate analyses showed that a low baseline creatinine level was the only significant predictor of response (Table 5).

**Table 5:** Comparison of baseline characteristics of responders vs nonresponders

Characteristic	Responders∗ (n = 28)	Nonresponders (n = 17)	*P*
Age, y	62.8 ± 10.6	62.8 ± 9.0	0.98
Male	24 (85.7)	16 (94.1)	0.63
BMI, kg/m^2^	24.4 [23.2–28.4]	28 [24.4 – 28.7]	0.22
OHCA	13 (46.4)	7 (41.2)	0.76
History of CAD	5 (17.9)	6 (35.3)	0.28
GRACE score, points	176 [162–198]	178 [155–189]	0.82
CardSHOCK score, points	3 [2–4]	2 [1–3.5]	0.41
New onset AKI	4 (15.4)	3 (17.6)	> 0.99
Creatinine at baseline, μmol/L	75 [57–86]	86 [76.5–97.5]	**0.018**
HR at implantation, bpm	92 ± 31	89 ± 24	0.72
LVEDP at implantation, mm Hg	33.4 ± 8.7	31 ± 11.3	0.54
Lactate at implantation	2 [1.4–3]	1.9 [1.5–4.9]	0.97
Peak troponin T, ng/L	7840 [4726–14547]	9760 [2631–15480]	0.99
Impella before PCI, n (%)	22 (78.6)	11 (64.7)	0.32
Impella support duration, hrs	32.3 [22.9–51.8]	25.1 [23.6–49.7]	0.87
LM as culprit vessel, n (%)	1 (3.6)	2 (11.8)	0.54
LVEF at baseline, %	32 ± 9	41 ± 14	**0.046**
LVEDDi at baseline, mm/m^2^	26.1 ± 3.9	24.3 ± 4.2	0.14
TAPSE at baseline < 17 mm	4 (14.3)	0 (0)	0.28
Three-pillars GDMT† at discharge	16 (57.1)	9 (52.9)	> 0.99
Four-pillars GDMT‡ at discharge	5 (17.9)	3 (17.6)	> 0.99
Loop diuretics at discharge	11 (39.3)	8 (47.1)	0.75

Values are n (%), mean ± standard deviation, or median [interquartile range]. Boldface indicates significance.

ACEi, angiotensin-converting enzyme inhibitors; AKI, acute kidney injury; ARB, angiotensin receptor blocker; ARNI, angiotensin receptor-neprilysin inhibitor; BMI, body mass index; bpm, beats per minute; CAD, coronary artery disease; CardSHOCK, GDMT, guideline-directed medical therapy; GRACE, Global Registry of Acute Coronary Events; HR, heart rate; LM, left main; LVEDDi, left ventricular diastolic diameter index; LVEDP, left ventricular end-diastolic pressure; LVEF, left ventricular ejection fraction; MRA, mineralocorticoid receptor antagonist; OHCA, out-of-hospital cardiac arrest; PCI, percutaneous coronary intervention; SGLT2i, sodium-glucose cotransporter 2 inhibitor; TAPSE, tricuspid annular plane systolic excursion.

∗ Defined as LVEF improvement ≥ 10% at follow-up.

† Defined as ACEi’s/ARBs/ARNIs + beta-blockers + MRAs.

‡ Defined as ACEi’s/ARBs/ARNIs + beta-blockers + MRAs + SGLT2i’s.

## Discussion

Anterior STEMI is commonly complicated by hemodynamic deterioration and cardiogenic shock, which in turn carry a high mortality risk.^15^, ^16^, ^17^ A major contributor to morbidity and mortality following an anterior STEMI is adverse cardiac remodelling.^18^ The use of the Impella device in selected anterior STEMI patients with shock presentation may improve short-term outcomes, but whether it has a beneficial long-term impact on cardiac remodelling remains unclear.^5^

In this context, our analysis highlights the following points:

• At long-term echocardiographic follow-up after anterior STEMI treated with the Impella device, most patients who survived the index event showed an improvement of LV function and stable parameters of ventricular dilation, diastolic function, and right ventricular function and dimension. Of note, 62% showed a prognostically significant ≥ 10% absolute increase of LVEF.
• Baseline creatinine level was the only significant predictor of response at univariate analysis.
• Compared to a median expected mortality of 50% at 6 months by means of the Global Registry of Acute Coronary Events-acute coronary syndrome (GRACE-ACS) score,^19^ our cohort showed a mortality rate of 26% at 6 months.

### Anterior STEMI and LV remodelling

In the contemporary era, in which STEMI is treated with primary PCI and optimal pharmacotherapy, almost one-half of patients demonstrate LV post-infarct remodelling.^20^ Given that post-MI heart failure has been shown to be associated with increased mortality,^14^^,^^21^^,^^22^ preventive measures limiting infarct size and counteracting LV remodelling are paramount in these patients.

With respect to infarct localization, anterior localization seems to be associated with a greater extent of microvascular obstruction and a larger infarct size, which results in a higher incidence of heart failure and adverse events (eg, arrhythmias, secondary mitral regurgitation).^15^, ^16^, ^17^ As a result, we decided to analyze the impact of an Impella device in a selected cohort of patients with anterior localization only, excluding patients with nonanterior infarction.

Guideline-based medical therapy (GDMT) for heart failure can mitigate adverse negative remodelling by reducing afterload, sympathetic drive, and fibrosis. Nevertheless, in our analysis, GDMT with or without sodium-glucose cotransporter 2 inhibitors was not a significant predictor of LVEF increase at univariate analysis, a result that is expected as the study was underpowered in sample size and length of follow-up time to anticipate this effect.

Whether LV unloading prevents adverse cardiac remodelling, or additionally enhances cardiac recovery, in patients presenting with acute MI complicated by cardiogenic shock remains unclear and certainly needs to be determined in further studies. To put our results into perspective, we summarized the data from earlier pivotal studies, which analyzed LV recovery following acute MI, as highlighted in Supplemental Table S7. Although LVEF recovery is generally seen with timely revascularization and GDMT in most of these earlierstudies that included acute MI patients, who mostly were not treated with MCS, one needs to consider that we analyzed a distinct and very sick cohort who presented with large anterior STEMI and were dependent on MCS. In our anterior STEMI cohort requiring Impella device support, an increase in LVEF of ≥ 10% was noted in almost two-thirds of all surviving patients.

### LV unloading for the prevention of heart failure

The Impella device has been hypothesized to have beneficial effects, via its counteracting of the detrimental impact of high wall stress and microvascular dysfunction following an anterior STEMI.

Reducing LV volume and LV end-diastolic pressure by mechanical unloading reduces myocardial oxygen demand, activates cardioprotective signaling cascades that mitigate myocardial damage after reperfusion, and may also promote myocardial recovery, which ultimately leads to a reduced infarct size.^7^^,^^8^ Furthermore, observational studies on cardiac magnetic resonance imaging suggested that LV unloading prevented cardiac dysfunction by preserving the global radial and circumferential strain, and the systolic and diastolic strain rates in the remote myocardium.^23^

As stated in the *Methods* section, the timing of Impella device insertion in our cohort followed mainly 2 approaches, depending on case-by-case clinical decision-making. Nevertheless, early evidence from animal models and observational trials seems to favour Impella device implantation before revascularization, which may maximize the potential benefit of LV unloading.^7^, ^8^, ^9^^,^^24^^,^^25^ After completion of a pilot feasibility study,^26^ the **STEMI-D**oor-**t**o-**U**nload (STEMI-DTU) trial is now ongoing to assess the safety and effectiveness of LV unloading with an Impella CP device for 30 minutes before PCI in patients with acute anterior STEMI (NCT03947619).

Our study provides real-world insight regarding the hypothesis that LV unloading could potentially counteract the impact of negative remodelling post-infarct, even in the long-term, irrespective of when LV unloading occurs, and facilitate subsequent LV recovery. Further studies are needed to better understand the mechanisms and utility of the Impella device in these processes.

### The role of right ventricular function

Right ventricular (RV) dysfunction after MI has a detrimental impact on prognosis.^27^^,^^28^ Anterior localization seems to be less commonly associated with RV failure, compared to other MI localizations in concomitant cardiogenic shock.^29^ This finding is confirmed in our cohort. On average, parameters of RV dimension and function at baseline were in the normal range, and only 4 patients had tricuspid annular plane systolic excursion < 17 mm. Interesting to note is that those patients were all responders at follow-up. In any case, the impact of RV failure on recovery and prognosis of cardiogenic shock patients receiving LV unloading with an Impella device is poorly described and warrants further investigation.

### Limitations

Our findings must be interpreted in the context of the following limitations. First, this is an observational single-centre study, which may limit its generalizability, and its results show association, not causation. Second, the analyzed echocardiograms were obtained in most cases as part of a routine clinical assessment and according to our local protocol. Due to clinical circumstances and/or limited availability of echocardiography staff, we encountered some variability in the timing of baseline and follow-up echocardiograms. As a result, the number of responders could have been underestimated due to a partial recovery before the first echocardiogram. Third, the limited number of patients in our cohort may have been insufficient to show statistical significance in some endpoints. Fourth, the absence of a control cohort of patients with anterior STEMI who were not treated with an Impella device or were implanted with other MCS devices limits the inferences that can be made about the impact of an Impella device alone, compared to usual care, on the endpoints. However, this issue is beyond the scope of our study. Moreover, selecting a control cohort that consisted of patients without any device support or who were implanted with other means of MCS would have introduced an uncontrolled bias, particularly as Impella implantation in patients who presented with anterior STEMI was made on a case-by-case basis by the treating invasive cardiologist.

## Conclusions

The present study highlights a positive trend in cardiac remodelling and function during the follow-up period of patients with acute anterior STEMI who received Impella device support on top of standard therapies including primary PCI. Eventually, the results from ongoing randomized trials are needed to determine whether hemodynamic support and LV unloading with Impella devices prevents adverse cardiac remodelling and the new onset of heart failure following acute anterior STEMI.
