# Supplementary material for: Trajectories of Cardiac Function Following Treatment With an Impella Device in Patients With Acute Anterior ST-Elevation Myocardial Infarction
Source: CJC Open. 2022 Nov 5;5(1):77–85. doi: 10.1016/j.cjco.2022.11.002 (PMC9869354; doi:10.1016/j.cjco.2022.11.002)

Supplementary Material

Trajectories of cardiac function following treatment with Impella in patients with acute anterior ST-elevation myocardial infarction

Supplemental Table S1. Baseline characteristics according to survival status.

	**Survivors** (n=50)	**Non-survivors** (n=16)
Age, years	62.6 (10.1)	69.7 (14.3)
Male, n (%)	44 (88.0)	15 (93.7)
BMI, kg/m^2	25.9 [23.8 – 28.7]	24.2 [24.2 – 26.1]
**Comorbidities**		
CAD, n (%)	11 (22)	1 (6.3)
Previous MI, n (%)	8 (16)	1 (6.3)
Previous PCI, n (%)	10 (20)	0
Previous CABG, n (%)	1 (2)	0
Heart failure, n (%)	2 (4)	0
Atrial fibrillation, n (%)	1 (2)	1 (6.3)
Previous stroke, n (%)	1 (2)	2 (12.5)
PAD, n (%)	5 (10)	2 (12.5)
CKD (eGFR <30 ml/min/1.73), n (%)	3 (6)	0
**Cardiovascular risk factors**		
Smoking, n (%)	25 (50)	6 (37.5)
Arterial hypertension, n (%)	26 (52)	8 (50)
Dyslipidemia, n (%)	22 (44)	3 (18.8)
Diabetes mellitus, n (%)	7 (14)	2 (12.5)
Family history of CAD, n (%)	9 (18)	2 (12.5)
**Clinical presentation**		
OHCA, n (%)	20 (40)	5 (31.3)
Profound cardiogenic shock, n (%)*	39 (78)	15 (93.4)
**SCAI class at admission**		
A, n (%)	11 (22)	1 (6.3)
B, n (%)	1 (2)	1 (6.3)
C, n (%)	6 (12)	2 (12.5)
D, n (%)	14 (28)	5 (31.3)
E, n (%)	18 (36)	7 (43.8)
GRACE score, points	178.24 (23.1)	206.75 (25.6)
CardSHOCK score, points	3 [2-4]	4 [4-4.75]

BMI, body mass index; CABG, coronary artery bypass graft surgery; CAD, coronary artery disease; CKD, chronic kidney disease; COPD, chronic obstructive pulmonary disease; eGFR, estimated glomerular filtration rate; MI, myocardial infarction; OHCA, out-of-hospital cardiac arrest; PAD, peripheral artery disease; PCI, percutaneous coronary intervention.
*defined as SCAI class C, D or E.

Supplemental Table S2. In-hospital management according to survival status.

	Survivors (n=50)	Non-survivors (n=16)
**Type of support**		
Impella 2.5, n (%)	3 (6)	0 (0)
Impella CP, n (%)	47 (94)	16 (100)
**Timing of support**		
Before PCI, n (%)	38 (74)	11 (68.8)
After PCI, n (%)	12 (24)	4 (25)
**Indication**		
Cardiac arrest, n (%)	28 (56)	9 (56.3)
Profound cardiogenic shock (SCAI class ≤C), n (%)	47 (94)	16 (100)
Beginning cardiogenic shock (SCAI class B), n (%)	3 (6)	0 (0)
LVEDP at implantation, mmHg	26.7 (9.1)	32.1 (9.6)
MAP at implantation, mmHg	66 [56 – 70]	71 [60 – 94.5]
Ongoing resuscitation during implantation, n (%)	3 (6)	6 (37.5)
Duration of support, hrs	31.4 [24 – 51.7]	34 [3.3 – 49.4]
PCI performed, n (%)	50 (100)	15 (93.8)
**Culprit vessel**		
LM, n (%)	4 (8)	9 (56.3)
LAD, n (%)	46 (92)	6 (37.5)
ICU stay, days	4 [2 – 10]	6 [1 – 14]
Mechanical ventilation, n	23 (46)	10 (62.5)
Length of mechanical ventilation, hrs	90 [40 – 192]	95.5 [19.3 – 240]
Inotropes/vasoactive drugs, n (%)	34 (68)	14 (87.5)
Duration of inotropes/vasoactives, hrs	60 [30 - 139]	48 [5 – 187]
New-onset AKI, n (%)	8 (16)	4 (25)
**Laboratory values**		
Hemoglobin at admission, g/L	139 [127.5 – 148.8]	128 [112 – 142.3]
Leucocytes at admission, G/L	12.4 [9.2 – 15.3]	13.6 [11.6 – 28.2]
Lactate at implantation, mmol/L	2.1 [1.4 – 3.5]	3.6 [2 – 11.9]
Creatinine at implantation, µmol/L	80 [65.5 – 94.3]	115 [95 – 136.5]

Peak creatinine, µmol/L	99 [83 – 141.5]	140 [116 – 495]
Troponin T at admissiont, ng/L	169 [38 – 495]	2346 [285 – 5229]
Peak troponin T, ng/L	9335 [4202–15484]	15563 [2221-31066]
Peak creatine kinase, U/L	5376 [3002–6857]	4418 [594–6662]
ALT, U/L	64 [37–158]	66 [28–268]

ALT, alanine aminotransferase; AKI, acute kidney injury; ICU, intensive care unit; LAD, left anterior descending artery; LM, left main; LVEDP, left ventricular end-diastolic pressure; MAP, mean arterial pressure; PCI, percutaneous coronary intervention.

Supplemental Table S3. Complications related to Impella implantation.

	**All patients (n=66)**
**Complications, n (%)**	**30 (45.5)**
Major bleeding, n (%)	*16 (24.2)*
Minor bleeding, n (%)	*7 (10.6)*
Vascular complication, n (%)	*11 (16.7)*
Vascular surgery, n (%)	*7 (10.6)*
Blood transfusion required, n (%)	*18 (27.3)*
Packed RBC transfused	*2.5 [1 – 6.75]*

RBC, red blood cells

Supplemental Table S4. Narrative compilation with details of relevant clinical data of patients with in-hospital death.

Patient no.	Time to death (days)	Presentation	Presumed cause of death	Culprit vessel	LVEF baseline (%)	AKI	ICU stay (days)
**1**	10	CS	Multiorgan failure	LAD prox.	31	X	9
**2**	12	CS	Refractory cardiogenic shock	LAD prox.	45		8
**3**	18	CS	Refractory cardiogenic shock	LAD prox.	20		14
**4**	6	CS	Refractory cardiogenic shock	LAD prox.	28	X	6
**5**	2	OHCA	Multiorgan failure	LM	-	X	2
**6**	0	CS	Refractory cardiogenic shock	LM	-		1
**7**	0	OHCA	Refractory resuscitation in cathlab	LM	-		0
**8**	44	CS	Therapy withdrawal because of poor neurological outcome	LM	30		14
**9**	1	CS	Refractory resuscitation in ICU	LAD prox.	10		1
**10**	0	CS	Refractory resuscitation in cathlab after PCI	LM	15		0
**11**	2	OHCA	Hemothorax and hemorrhagic shock	LM	-		3
**12**	32	CS	Refractory resuscitation in ward	LAD prox.	15	X	26
**13**	1	OHCA	Brain death	LM	15		1
**14**	44	CS	Sepsis and toxic epydermolysis in another hospital	LM	35	X	15
**15**	0	OHCA	Refractory resuscitation in cathlab before PCI	LAD prox.	-		0
**16**	13	OHCA	Therapy withdrawal because of poor neurological outcome	LAD mid	43		10

AKI, acute kidney injury; CS, cardiogenic shock; ICU, intensive care unit; LAD, left anterior descending artery; LM, left main; LVEF, left ventricular ejection fraction; OHCA, out-of-hospital cardiac arrest; PCI, percutaneous coronary intervention.

Supplemental Table S5. Therapy at hospital discharge after index hospitalization.

	Discharged patients *(n=50)*
Therapy at discharge	
ACE-Inhibitors, n (%)	*37 (74%)*
ARBs, n (%)	*3 (6%)*
ARNI, n (%)	*7 (14%)*
Beta-blockers, n (%)	*41 (82%)*
MRA, n (%)	*33 (66%)*
SGLT2i, n (%)	*8 (16%)*
Oral anticoagulants, n (%)	*7 (14%)*
DAPT, n (%)	*48 (96%)*
Loop diuretics, n (%)	*23 (46%)*

ACE, angiotensin-converting enzyme; ARB, angiotensin receptor blockers; ARNI, angiotensin receptor-neprilysin inhibitors; DAPT, dual antiplatelet therapy; MRA, mineralocorticoid receptor antagonists; SGLT2i, sodium-glucose cotransporter 2 inhibitors

Supplemental Table S6. Echocardiographic parameters of survivors (n=50) and non-survivors (n=16) at baseline.

	Survivors (n=50)	Non-survivors (n=16)	p-value
LVEF, %	36±11 (n=50)*	26±12 (n=11)	**0.02**
LVEDD, mm	48.0 [45.0 – 52.0] (n=48)	48.2±9.4 (n=5)	0.67
LVEDDi, mm/m²	25.5±4.1 (n=48)	25.3 [23.7– 29.7] (n=5)	0.52
E/e’	9.6 [8.0 – 11.6] (n=30)	21.75 [9.1 – 34.4] (n=2)	N/A
LAVi, ml/m²	28.0 [23.0 – 43.0] (n=26)	27.5 (n=1)	N/A
TAPSE, mm	22 [19 – 24] (n=45)	22 [19 – 22] (n=4)	0.81
RV S’, cm/s	15.6±4.2 (n=34)	14.3 (n=1)	N/A
TR Vmax, m/s	2.8 [2.4 – 2.8] (n=18)	3.4 [2.7 – 4.2] (n=2)	N/A
RVEDAi, cm2/m²	9.2 [7.0 – 10.5] (n=9)	12.0 (n=1)	N/A

** Number of recorded observations.*

LAVi, left atrial volume index; LVEDD, left ventricular diastolic diameter; LVEDDi, left ventricular diastolic diameter index; LVEF, left ventricular ejection fraction; RVEDAi, right ventricular end-diastolic area index; RV S’, right ventricular systolic excursion velocity; TAPSE, tricuspid annular plane systolic excursion; TR Vmax, tricuspid regurgitation peak jet velocity.

Supplemental Table S7. Pivotal studies describing long-term recovery of left ventricular ejection fraction following acute myocardial infarction, sorted by increasing follow-up time.

Study	N	Patient cohort	Anterior MI*	LVEF baseline (%)	LVEF at FU (%)	(mean) Follow-up time
**CARISMA[1]**	312	AMI with LVEF < 40%	56%	31±6	35±10	6 weeks
**REFINE[2]**	322	AMI with LVEF < 40% in the first 48h or < 50% after the first 48h	62%	40 (35 – 44)	47 (38 – 55)	8-10 weeks
**Savoye, 2006[3]**	215	Anterior AMI	100%	49.6±9.4	51.3±9.2	3 months
**van der Bijl, 2019[4]**	1995	STEMI	44%	47±9	51±10	3 months
**Sjöblom, 2014[5]**	91	AMI with LVEF < 40%	65%	31± 5.8	40±11	3 months
Present study	*65*	*Acute anterior STEMI treated with Impella*	*100%*	*36.2±11.2*	*48.0±13.2*	*4 months*
**Parodi, 2007[6]**	228	AMI	72%	38±7	47±12	6 months
**Elsman, 2006[7]**	432	LAD-related AMI	100%	39±11	43±11	6 months
**Park, 2021[8]**	4008	AMI	Unknown	51.6±10.8	55.5±10.7	12 months

AMI, acute myocardial infarction; FU, follow-up; LAD, left anterior descending artery; LM, left main; LVEF, left ventricular ejection fraction; MI, myocardial infarction; STEMI, ST-elevation myocardial infarction.

* *Defined as "anterior location" or "LAD/LM related AMI".*

REFERENCES

1. Huikuri HV, Raatikainen MJP, Moerch-Joergensen R, et al. Prediction of fatal or near-fatal cardiac arrhythmia events in patients with depressed left ventricular function after an acute myocardial infarction. *European Heart Journal*. 2008;30(6):689-698. doi:10.1093/eurheartj/ehn537
2. Exner DV, Kavanagh KM, Slawnych MP, et al. Noninvasive Risk Assessment Early After a Myocardial Infarction. *Journal of the American College of Cardiology*. 2007;50(24):2275-2284. doi:10.1016/j.jacc.2007.08.042
3. Savoye C, Equine O, Tricot O, et al. Left Ventricular Remodeling After Anterior Wall Acute Myocardial Infarction in Modern Clinical Practice (from the REmodelage VEntriculaire [REVE] Study Group). *The American Journal of Cardiology*. 2006;98(9):1144-1149. doi:10.1016/j.amjcard.2006.06.011
4. van der Bijl P, Abou R, Goedemans L, et al. Left Ventricular Post-Infarct Remodeling. *JACC: Heart Failure*. 2020;8(2):131-140. doi:10.1016/j.jchf.2019.08.014
5. Sjöblom J, Muhrbeck J, Witt N, Alam M, Frykman-Kull V. Evolution of Left Ventricular Ejection Fraction After Acute Myocardial Infarction: Implications for Implantable Cardioverter-Defibrillator Eligibility. *Circulation*. 2014;130(9):743-748. doi:10.1161/CIRCULATIONAHA.114.009924
6. Parodi G, Memisha G, Carrabba N, et al. Prevalence, Predictors, Time Course, and Long-Term Clinical Implications of Left Ventricular Functional Recovery After Mechanical Reperfusion for Acute Myocardial Infarction. *The American Journal of Cardiology*. 2007;100(12):1718-1722. doi:10.1016/j.amjcard.2007.07.022
7. Elsman P, van‘t Hof AWJ, de Boer MJ, et al. Impact of infarct location on left ventricular ejection fraction after correction for enzymatic infarct size in acute myocardial infarction treated with primary coronary intervention. *American Heart Journal*. 2006;151(6):1239.e9-1239.e14. doi:10.1016/j.ahj.2005.12.006
8. Park CS, Yang HM, Ki YJ, et al. Left Ventricular Ejection Fraction 1 Year After Acute Myocardial Infarction Identifies the Benefits of the Long-Term Use of β-Blockers: Analysis of Data From the KAMIR-NIH Registry. *Circ: Cardiovascular Interventions*. 2021;14(4):e010159. doi:10.1161/CIRCINTERVENTIONS.120.010159

Supplemental Figure S1. Left ventricular ejection fraction (LVEF) at baseline and first follow-up among survivors.

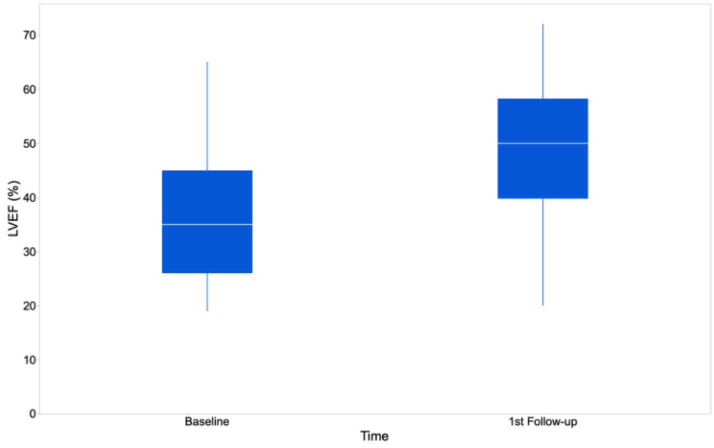

Supplemental Figure S2. Left ventricular end-diastolic diameter (LVEDD) at baseline and first follow-up among survivors.

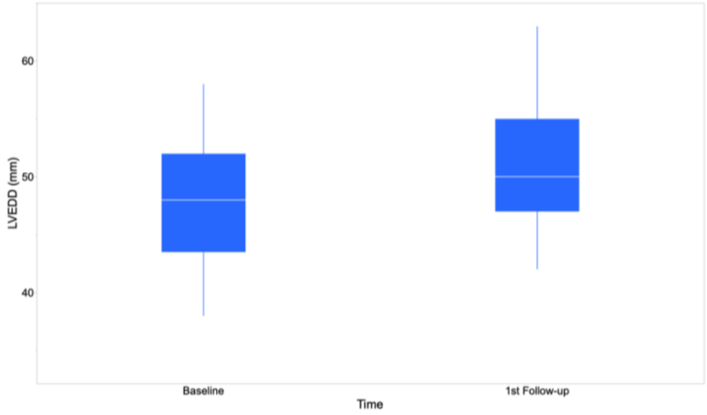

Supplemental Figure S3. E/e' at baseline and first follow-up among survivors.

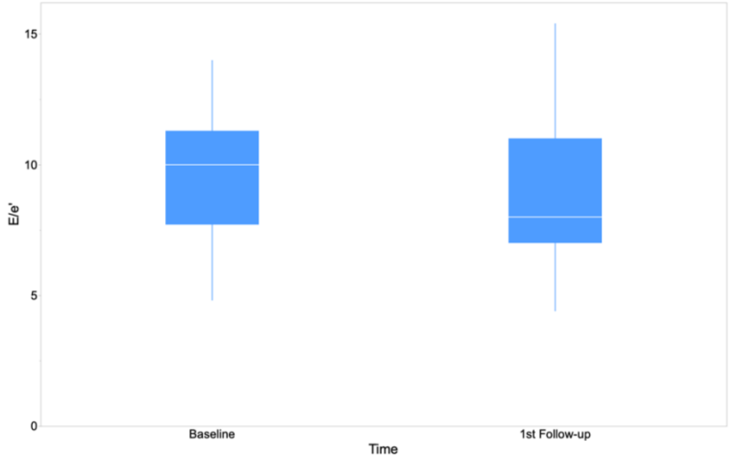

Supplement: Supplementary Material [file mmc1.pdf]
